# Concentrations of Fat, Protein, Lactose, Macro and Trace Minerals in Alpaca Colostrum and Milk at Different Lactation Stages

**DOI:** 10.3390/ani11071955

**Published:** 2021-06-30

**Authors:** Maria Mößler, Janina Aichner, Anja Müller, Thiemo Albert, Thomas Wittek

**Affiliations:** 1 Department for Farm Animals and Veterinary Public Health, University of Veterinary Medicine Vienna, Veterinärplatz 1, 1210 Vienna, Austria; janina92@outlook.com (J.A.); Thomas.Wittek@vetmeduni.ac.at (T.W.); 2 Vet Med Labor GmbH, IDEXX Laboratories, Mörikestraße 28/3, 71636 Ludwigsburg, Germany; Anja-Elvira-Mueller@idexx.com; 3 Institute of Food Hygiene, Veterinary Faculty at Leipzig University, An den Tierkliniken 1, 04103 Leipzig, Germany; albert@vetmed.uni-leipzig.de

**Keywords:** alpaca, colostrum, composition, fat, lactose, milk, minerals, protein

## Abstract

**Simple Summary:**

Alpacas and llamas are domesticated species of New World camels. If the mare dies or produces insufficient colostrum or milk, information about the composition of colostrum and milk is needed to formulate suitable substitutes to adequately supply the crias. Milk composition in alpacas has been sparsely studied. In this study colostrum samples were taken daily during the first four days after parturition and milk samples were obtained monthly during the first four months of lactation. The samples were analyzed for their composition. The fat and lactose content are lowest on the day of birth and then increase, the protein content decreases during the first four days. Over the next four months, these contents do not change significantly. The results can be used for the development of colostrum and milk replacers.

**Abstract:**

Although alpacas are not used for milk production a detailed knowledge on the composition of the colostrum and milk is needed for development of colostrum and milk replacers. The aim of the present study was to measure the concentration of fat, protein, lactose, and minerals in alpaca colostrum and milk. Colostrum samples were taken daily over four days after parturition from 20 multiparous alpaca mares. Milk samples were obtained monthly, during the first four months of lactation from 17 alpacas. Composition of colostrum and milk differed in numerous indicators. The concentrations of fat and lactose increased from day 1 (0.5%, 4.0%) to day 4 (5.3%, 5.0%), protein decreased from 20.4% on day 1 to 8.3% on day 4. In milk these three indicators did not change during the lactation. Minerals have been little studied in alpaca colostrum and milk in the past, many of which had the highest concentrations in colostrum immediately after birth. The results of the present study do not support that goat’s milk is the preferred substitute for feeding crias. This study contributes to the knowledge of the composition of alpaca colostrum and milk which can be of particular use in developing replacers.

## 1. Introduction

Alpacas, along with llamas, are one of the domesticated species of New World camelids [1,2]. Although alpacas and llamas are not used for milk production knowledge on the composition of the colostrum and milk is needed for development of colostrum and milk replacers. Such replacers are required for crias which have to be hand reared if their mothers have died or are not producing sufficient quantity of milk. Currently milk from other species, mostly cattle or goat or milk replacers designed for calves, lambs, and kids are used [3].

In contrast to numerous studies in several dairy species only a small number of studies have been reported on the composition of milk from South American camelids (SAC) [4,5,6,7,8,9]. The studies on colostrum composition in alpacas have mainly reported on immunoglobulin concentration [10,11,12]. Additionally, studies on alpaca which is a dominant SAC breed in many countries are mostly based on a small number of animals [13,14,15].

One study measured colostrum constituents taking samples 48 h after parturition, comparing alpacas kept in different altitudes (18 animals at sea level and 24 animals at 4400 m above sea level). Later in the same study milk samples were obtained monthly from the first into the fifth month of lactation. However, to obtain sufficient volume for analyses (60 mL) the researchers had to pool the samples of three animals, reducing the effective number of samples by a third. Colostrum fat, protein, and lactose concentrations did not differ between the habitats, but milk constituents were influenced by lactation status [16]. The content of fat, protein, casein, and minerals (calcium, phosphorus, magnesium, potassium, sodium, and zinc) were measured in the milk of eight alpaca mares taken on the 30th and 60th lactation day in Italy [17]. Chad [18] studied the composition of alpaca milk (calcium, phosphorus, potassium, magnesium, sodium, sulfur, lactose, fat, protein, and urea) using milk samples of 11 alpaca mares taken on two Californian farms. The samples were taken every week during the first 25 weeks of lactation. To our best knowledge studies on trace elements in colostrum or milk from SAC have not been published so far.

The aims of the present study were to measure the concentration of fat, protein, lactose, macro and trace minerals in alpaca colostrum and milk at different stages of lactation. Further the composition of colostrum and milk should be compared to information given in the literature for SAC and other species.

It was hypothesized that:

1. The composition of colostrum from alpacas changes within the four day colostral period after parturition.
2. The composition of milk changes according to the lactation status in alpaca mares.
3. The composition of alpaca colostrum and milk differs in certain indicators from ruminant milk which supports the development of species specific colostrum and milk replacers.

## 2. Materials and Methods

### 2.1. Animals and Sampling Procedures

The project was discussed and approved by the institutional ethics and animal welfare committee in accordance with GSP guidelines and national legislation (Ethic Code ETK-21/11/2016).

#### 2.1.1. Alpacas for Colostrum Sampling

The colostrum samples were taken from 20 alpaca mares kept in three smaller alpaca farms in the district Bruck-Mürzzuschlag in Styria (Austria). The farms were in close proximity having almost identical weather, husbandry, and feeding conditions. All available pregnant multiparous alpaca mares of the foaling season (June–September) were included in the study. The animals belonged to the Huacaya breed and were between four and nine years old. They were pastured in the Austrian Alps at an altitude between 700 and 1100 m above sea level on a calcareous source rock. The animals were additionally offered hay ad libitum. Colostrum samples were taken on four consecutive days after foaling, the first samples (day 1) were taken on the day of birth between two and four hours after parturition. Crias were not separated from their mothers, but the teats were closed with a tape at least two hours before sampling. After that, the entire amount of colostrum was milked by hand.

#### 2.1.2. Alpacas for Milk Sampling

The milk samples were obtained from lactating alpacas four times at monthly intervals from all 17 alpaca mares on a farm in the Alps in South Tyrol (Northern Italy). Mothers and crias were not separated the whole time, but only for two hours before sampling. These animals (15 Huacaya and two Suri) were comprised of ages between three and 10 years. Thirteen of the mares were multiparous and four had given birth to their first cria. The alpacas grazed on alpine meadows between 1320 and 1550 m above sea level on a calcareous source rock.

#### 2.1.3. Sample Preparation

For sampling the mares were restrained by their owners using a halter and were milked by hand. The udders were examined for conspicuous redness, swelling, and induration to exclude animals with clinical mastitis. Colostrum or milk from all four udder quarters was obtained in similar volume. Samples obtained were examined for visible milk changes that would indicate mastitis. After milking the sample container was immediately transferred to a cooled polystyrene box (4 °C) (Henry Schein Medical Austria GmbH, Vienna, Austria). Within one hour the samples were divided into sub samples in Eppendorf^®^ Safe-Lock microcentrifuge tubes (volume 2.0 mL, Eppendorf Austria GmbH, Vienna, Austria) and in one Greiner tube (15.0 mL volume, Greiner Bio-One GmbH, Kremsmünster, Austria) and stored at −18 °C and shipped to the laboratories.

### 2.2. Laboratory Analyses

#### 2.2.1. Analyses of Milk Fat, Protein, and Lactose Concentration

Analyses of milk and colostrum fat, lactose, and protein concentrations were performed at the laboratory of the Institute of Food Hygiene, Veterinary Faculty at Leipzig University applying standardized laboratory methods as described by the German Industry Standard (Deutsche Industry Norm, DIN). The fat content was determined using the Weibull–Berntrop gravimetric method [19]. Due to the limited volume of colostrum or milk the analysis of fat concentration could only be performed as a single measurement. The content of lactose was analyzed by the UV lactose/D-galactose method (Roche Diagnostics^®^, Mannheim, Germany) and the protein content by the Kjehldahl method [20].

#### 2.2.2. Analyses of Macro and Trace Minerals

The milk and colostrum samples were analyzed at the Vet Med Labor GmbH, IDEXX Ludwigsburg (Germany). The elements sulfur (S), phosphorus (P), sodium (Na), and potassium (K) were analyzed by inductive coupled plasma optical emission spectrometry (ICP-OES) using Vista-Pro device (Varian Inc., Palo Alto, CA, USA) and the elements lithium (Li), boron (B), magnesium (Mg), aluminum (Al), calcium (Ca), manganese (Mn), iron (Fe), cobalt (Co), nickel (Ni), copper (Cu), zinc (Zn), arsenic (As), selenium (Se), strontium (Sr), molybdenum (Mo), cadmium (Cd), tin (Sn), barium (Ba), thallium (Tl), lead (Pb), and uranium (U) by the inductive coupled plasma mass spectrometry (ICP-MS) device Aurora M90 (Bruker Daltonics, Bremen, Germany). The coefficients of variation of the analyses are shown in Table 1 for each measured parameter.

### 2.3. Statistical Analyses

Since not all data were normally distributed (Kolmogorov–Smirnov Test), all the indicators are presented as median and first/third quartile. Composition of milk and colostrum was compared using the nonparametric Mann–Whitey U Test. Further analyses were performed using log transformed data. The fat, protein, lactose, and mineral element concentrations were compared over time separately for colostrum and milk using a mixed linear model (measurement repetition). The individual alpaca mare was considered as a random effect, while the age of the animal, the day in milk and the lactation number were fixed effects. The Bonferroni test was applied as a post-hoc test. Microsoft Excel 2010 and the SPSS Statistics Version 24 (IBM Corp., Armonk, NY, USA) were used for statistical analysis. The significance level was set at 5%.

## 3. Results

### 3.1. Colostrum and Milk Volume

A total of 77 colostrum samples were obtained from the 20 mares during four days postpartum. The three missing samples were due to one animal dying on day 3 and one animal developed a clinical mastitis on day 4. The volumes which could be obtained varied between 12 and 28 mL with a median of 20.8 mL. There was no difference between the days in the obtainable colostrum volume.

Overall, 61 milk samples were obtained (17 in the first month, 16 in the second month, 16 in the third month, 12 in the fourth month). The hand milking of the alpaca mares became generally more difficult with increasing duration of lactation and in some animals in advanced lactation only very small volumes or no milk at all could be obtained. One animal had to be excluded since it developed a clinical mastitis in lactation month 2, while in four animals no milk could be obtained in month 4. The volumes which could be obtained varied between 0 and 25 mL with a significant difference between the milk volumes of the months 1 and 2 (median 14.0 mL and 12.5 mL) and the volumes of month 3 and 4 (median 8.0 mL and 5.5 mL).

### 3.2. Fat, Protein, and Lactose Concentrations in Colostrum and Milk

**Figure 1:**
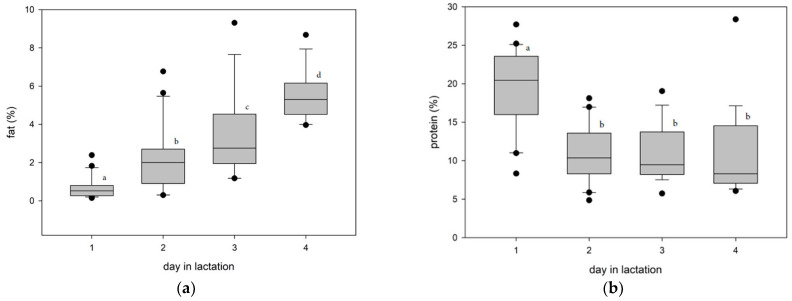

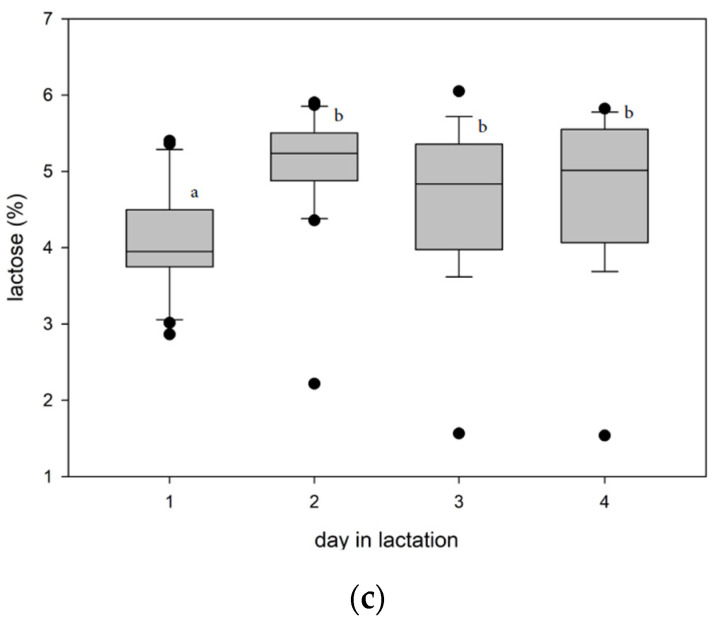
(**a**) Boxplot of fat concentration in alpaca colostrum during four days postpartum, significant differences between the days are marked with different indices. (**b**) Boxplot of protein concentration in alpaca colostrum during four days postpartum, significant differences between the days are marked with different indices. (**c**) Boxplot of lactose concentration in alpaca colostrum during four days postpartum, significant differences between the days are marked with different indices.

**Figure 2:**
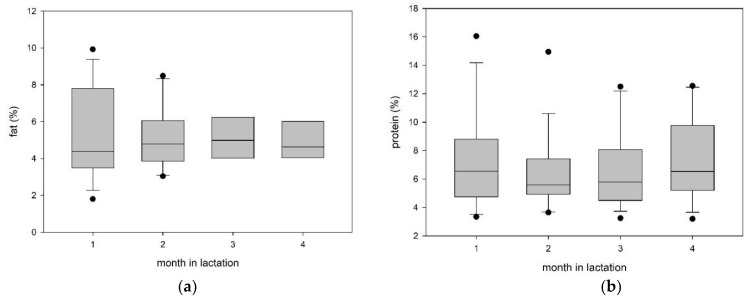

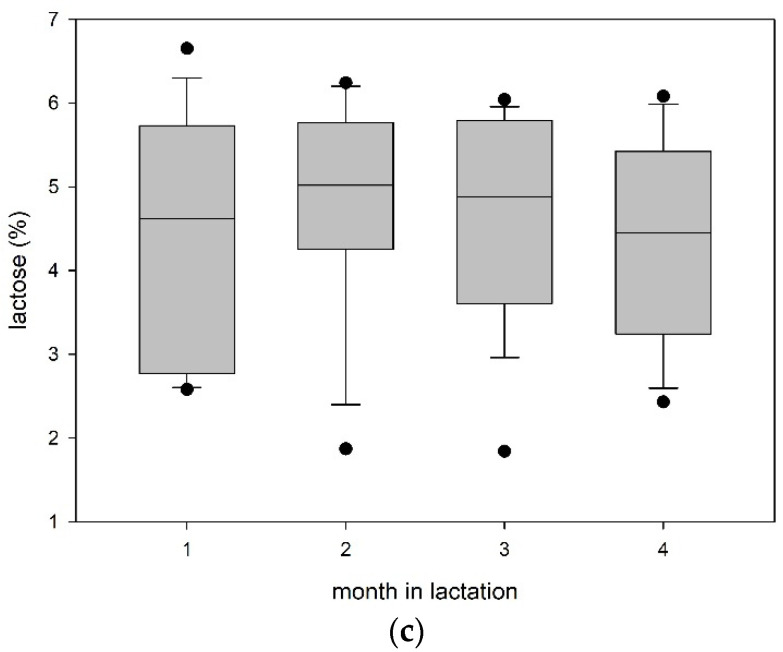
(**a**) Boxplot of fat concentration in alpaca milk during four months postpartum, there were no significant differences between the months. (**b**) Boxplot of protein concentration in alpaca milk during four months postpartum, there were no significant differences between the months. (**c**) Boxplot of lactose concentration in alpaca milk during four months postpartum, there were no significant differences between the months.

The colostrum concentrations of fat, protein, and lactose are shown in Figure 1 and Table 2. The concentration of fat in colostrum increased significantly from day 1 to day 4; at day 4 the fat concentration was already similar to the fat concentration in milk during later lactation (Figure 2, Table 3). The lactose concentration on the day of parturition (day 1) was lower in comparison to days 2, 3, and 4. In contrast the colostrum protein concentration decreased substantially over the colostral period; however, the protein concentration on day 4 was significantly higher in comparison to the concentrations in milk later during lactation (Figure 2, Table 3).

The concentrations of milk fat, protein and lactose during four months into lactation are shown in Figure 2 and Table 3. None of the three indicators differed over the sampling period.

### 3.3. Mineral Concentrations in Colostrum and Milk

The concentrations of macro and trace elements are presented in Table 4 (colostrum) and Table 5 (milk). The concentration of Ca, P, and Mg decreased during the colostral period, having the highest concentration at the day of parturition (day 1). The same condition was present in a number of trace elements (Fe, Cu. Zn, Sr, Ba, Co, Ni, S) showing the highest concentrations immediately after parturition (Table 4).

During later lactation, milk calcium was the only macro element for which concentrations decreased during the four months of lactation. A comparison of mineral concentration between colostrum and milk is shown in Table 6. The concentration of numerous macro and trace elements differ significantly. Additionally, for comparison upper concentration limits for drinking water (Austria) and concentration for llama and alpaca milk reported in the literature are provided.

## 4. Discussion

As hypothesized, the composition of fat, protein, and lactose concentration in colostrum changed substantially during the colostral period. The decreasing concentration of protein and the increase of fat concentration mirrored that of other animal species [21,22,23]. The substantial decrease in colostral protein concentration can be attributed to the decrease in immunoglobulin concentration as described by Mößler [24] in alpacas and occurs in most other mammals [25,26,27,28,29,30]. For comparison Parraguez [16] took colostrum samples 48 h after parturition from alpaca mares in two regions of Chile. Six pooled alpaca colostrum samples taken in a herd kept at sea level in Patagonia had concentrations of 2.71 ± 0.6% fat, of 9.24 ± 0.5% protein, and of 5.33 ± 0.1% lactose whereas in eight pooled samples from the High Altiplano region (4400 m above sea level) concentrations of 4.80 ± 1.2% fat, of 9.84 ± 0.6% protein, and of 4.41 ± 0.1% lactose were measured. These measurements at 48 h after parturition are comparable and similar to the measurements at day 2 and 3 of the present study. The concentration of fat and lactose in colostrum at day 1 of the present study was also similar to the concentration measured in llama colostrum (fat 0.75 ± 0.25%, lactose 4.12 ± 0.46%) taken between four and 12 h after parturition [8]. However, the protein concentration in alpaca colostrum in the present study appeared to be higher in comparison to llamas at the same time (16.79 ± 1.64%) [8]; since the sample sizes are rather small in both studies (20 alpacas and nine llamas) it is impossible to draw conclusions on species differences. The composition of colostrum milked at day 4 after parturition is similar considering numerous indicators to milk later during lactation.

Chad [18] measured on average 3.68 ± 1.32% fat, 4.53 ± 0.78% protein and 6.00 ± 0.48% lactose in milk of 11 alpacas over 25 weeks into lactation. The minor differences to the present study might be due to the fact that they also included animals in the first week of lactation meaning they used some colostrum samples and they applied a different method (infrared spectrometry) for measurement which has been validated for cow milk. In a study covering five months of lactation performed in two regions of Chile Parraguez [16] found in six pooled alpaca milk samples in Patagonia (sea level) concentrations of 2.6 ± 0.5% fat, of 6.5 ± 0.3% protein and of 5.2 ± 0.5% lactose and in eight pooled samples from High Altiplano (4400 m above sea level) concentrations of 3.8 ± 0.6% fat, of 6.9 ± 0.3% protein and of 4.4 ± 0.5% lactose. The milk composition in Chile was also similar to the present study.

In contrast in comparison to llama milk in which Morin [7] in the USA found a fat concentration of 2.7 ± 1.0%; the fat concentration in alpaca milk in the present study was substantially higher. Additionally, the reported lactose (6.5 ± 0.5%) and protein (3.4 ± 0.4%) concentrations in lamas in the study by Morin [7] also differed substantially from the indicators in the present study in alpacas. Since the used laboratory methods were similar possible reasons for the differences are not obvious, however the diet might have had a substantial influence. The results for llama milk composition of a German study [8] (fat 4.70 ± 0.81%, protein 4.23 ± 0.23%, lactose 5.93 ± 0.27%) and an Argentinian study [9] (fat 4.55 ± 0.66%, protein 4.33 ± 0.17%, lactose 6.34 ± 0.34%) were much closer to the results of the present study in alpacas.

Comparing fat, lactose, and protein concentration of alpaca milk to goat milk (fat 3.9%, protein 3.3%, lactose 4.2%) and cow milk (fat 3.8%, protein 3.4%, lactose 4.7%) it seems that there is no firm rationale for the widely accepted opinion in the breeder community that goat’s milk is the preferred option for feeding alpaca crias [31]. Our clinical experience also does not support this.

The mineral content of alpaca colostrum and milk found in the present study is difficult to compare to other studies in SAC. It seems that there are no studies available on the mineral content of SAC colostrum. Further, it was observed that minerals are obviously stored in the udder before parturition as the concentration of a number of elements was highest in the first colostrum and decreased within the colostral period (Table 4). Only sparse information is available especially for trace elements in milk (Table 5). In a study by Morin [7] in 1995 the concentrations of a number of trace elements (B, Co, Mo, Sn, As, Cr, Cd, Hg, Pb, Se, Tl) were below the detection limits of the methods used at that time. Over 25 years later these technical limitations no longer exist; however, comparisons between different measurement methods are always difficult to draw.

Additionally it has to be considered that the trace element concentrations found in colostrum and milk are influenced by the availability of those minerals in feed. The study found some substantial differences in a number of elements between colostrum and milk. However, since the alpacas herds in which colostrum and milk samples were obtained are kept in different areas of the Alps with similar but not identical conditions it remains unclear which part of the differences is caused by the lactation status or by the different feed supply.

A limitation of the present study was that the trace and macro minerals were studied only in two regions in the Alps which were geologically similar. Since the source rock has an influence on the mineral content of milk, the results may therefore differ in other regions with different geological conditions. Further studies in geologically different areas on larger sample sizes would provide more reliable data.

Summarizing the findings, the present study contributed to the establishment of reference ranges for these indicators, albeit there were some differences between fat, protein, lactose, and macro element concentrations between the present study and information from different literature sources. Taken together, they provide guidance for replacement colostrum and milk from other sources including milk replacers for motherless reared crias.

## 5. Conclusions

The components fat, protein, and lactose change significantly in the first four days of lactation and remain at a constant level during the further months of lactation. Concentrations of numerous macro and trace elements also differ significantly over lactation especially during the first four days after parturition. The composition of colostrum and milk is substantially different from cow or goat milk. It appears that there is no rationale for the widely accepted opinion that goat milk is the preferred option for feeding motherless alpaca crias.

## Figures and Tables

**Table 1:** Coefficients of variation (CV) for the analysis of macro and micro minerals.

**Mineral**	**P** [%]	**Ca** [%]	**Mg** [%]	**Na** [%]	**K** [%]	**S** [%]		**Fe** [%]	**Cu** [%]	**Zn** [%]	**Se** [%]	**Sr** [%]	**Mo** [%]
CV	4.7	4.7	3.9	12.0	1.0	11.0		3.4	8.0	6.2	11.0	4.2	5.2
**Mineral**	**Ba** [%]	**Al** [%]	**Co** [%]	**Ni** [%]	**Tl** [%]	**Pb** [%]	**U** [%]	**Cd** [%]	**Sn** [%]	**As** [%]	**Mn** [%]	**B** [%]	**Li** [%]
CV	3.6	8.5	2.6	3.6	2.8	3.2	11.7	5.9	12.4	5.2	3.0	3.9	9.9

**Table 2:** Percentages of fat, protein, and lactose in alpaca colostrum during the four days postpartum, data are provided as median (1st quartile/3rd quartile); significant differences between the days are marked with different indices.

Colostrum	Day 1	Day 2	Day 3	Day 4
	(*n* = 20)	(*n* = 20)	(*n* = 19)	(*n* = 18)
Fat [%]	0.51 (0.29/0.77)^a^	2.01 (0.95/2.53)^b^	2.78 (2.03/4.52)^c^	5.31 (4.57/5.95)^d^
Protein [%]	20.4 (16.6/23.3)^a^	10.4 (8.50/13.4)^b^	9.48 (8.24/13.6)^b^	8.30 (4.13/13.5)^b^
Lactose [%]	3.95 (3.78/4.49)^a^	5.23 (4.92/5.46)^b^	4.84 (4.17/5.31)^b^	5.01 (4.13/5.51)^b^

**Table 3:** Percentages of fat, protein and lactose in alpaca milk during four months postpartum, data are provided as median (1st quartile/3rd quartile); there were no significant differences between the months.

Milk	Day 6–30 (*n* = 17)	Day 31–60 (*n* = 16)	Day 61–90 (*n* = 16)	Day 91–120 (*n* = 12)	
Fat [%]	4.39 (3.57/7.33)	4.78 (3.92/5.76)	4.98 (4.25/5.89)	4.64 (4.08/6.05)
Protein [%]	6.55 (4.86/8.36)	5.59 (4.94/7.21)	5.79 (4.75/7.38)	6.53 (5.34/9.68)
Lactose [%]	4.62 (3.32/5.47)	5.02 (4.31/5.73)	4.88 (3.74/5.78)	4.45 (3.45/5.39)

**Table 4:** Concentrations of macro and trace elements in alpaca colostrum during the four days postpartum, data are provided as median (1st quartile/3rd quartile), significant differences between the days are marked with different indices.

Colostrum	Day 1 (*n* = 20)	Day 2 (*n* = 20)	Day 3 (*n* = 19)	Day 4 (*n* = 18)
P [g/L]	2.51 (2.01/2.96)^a^	1.94 (1.77/2.01)^b^	1.80 (1.63/1.91)^b,c^	1.64 (1.55/1.75)^c^
Ca [g/L]	1.96 (1.74/2.34)^a^	1.55 (1.35/1.76)^b^	1.60 (1.43/1.86)^b^	1.48 (1.29/1.89)^b^
Mg [g/L]	0.60 (0.47/0.66)^a^	0.19 (0.17/0.32)^b^	0.21 (0.15/0.27)^b^	0.36 (0.29/0.39)^b^
Na [g/L]	0.36 (0.29/0.39)	0.37 (0.32/0.43)	0.43 (0.36/0.64)	0.40 (0.32/0.55)
K [g/L]	1.28 (1.19/1.45)	1.26 (1.10/1.52)	1.34 (0.86/1.46)	1.20 (0.93/1.32)
S [g/L]	2.10 (1.86/2.34)^a^	1.10 (0.92/1.45)^b^	1.04 (0.92/1.54)^b^	0.87 (0.74/1.45)^b^
Fe [mg/L]	0.88 (0,74/1.03)^a^	0.68 (0.58/0.81)^b^	0.72 (0.59/0.90)^a,b^	0.79 (0.64/0.95)^a,b^
Cu [mg/L]	0.48 (0.43/0.62)^a^	0.49 (0.41/0.73)^a^	0.64 (0.49/0.73)^a,b^	0.78 (0.51/1.15)^b^
Zn [mg/L]	7.20 (5.76/9.43)^a^	5.62 (4.90/6.20)^b^	3.83 (3.12/5.32)^b^	3.41 (2.70/3.86)^c^
Se [µg/L]	189 (125/304)^a^	104 (80.8/129)^b^	113 (78.6/158)^b^	87.1 (69.1/127)^b^
Sr [mg/L]	1.78 (1.14/1.97)^a^	1.12 (1.02/1.42)^a,b^	1.14 (1.01/1.48)^a,b^	1.07 (0.81/1.45)^b^
Mo [µg/L]	9.35 (6.55/13.4)	7.40 (5.00/12.2)	8.70 (6.8/18.4)	7.50 (3.50/14.8)
Ba [µg/L]	1006 (594/1255)^a^	658 (417/788)^a,b^	661 (434/885)^a,b^	431 (349/985)^b^
Al [µg/L]	29.0 (23.1/60.6)	44.2 (34.5/75.7)	52.8 (33.9/69.3)	38.6 (24.4/57.4)
Co [µg/L]	6.76 (4.08/9.66)^a^	1.92 (1.61/3.30)^b^	2.12 (0.93/3.12)^b^	0.85 (0.66/2.88)^b^
Ni [µg/L]	8.42 (7.51/9.74)^a^	6.53 (5.60/7.58)^b^	6.86 (6.60/8.31)^a,b^	6.12 (5.59/8.14)^b^
Tl [µg/L]	0.14 (0.13/0.21)	0.12 (0.09/0.19)	0.09 (0.06/0.14)	0.08 (0.05/0.11)
Pb [µg/L]	0.97 (0.48/2.01)	1.23 (0.82/1.56)	0.98 (0.74/1.43)	1.00 (0.78/1.60)
U [µg/L]	0.01 (0.01/ 0.04)	0.08 (0.04/0.13)	0.06 (0.01/0.23)	0.04 (0.02/0.10)
Cd [µg/L]	0.10 (0.00/0.16)	0.07 (0.00/0.17)	0.06 (0.01/0.23)	0.05 (0.00/0.15)
Sn [µg/L]	0.80 (0.40/1.39)	0.60 (0.42/1.11)	0.83 (0.51/1.93)	0.82 (0.49/1.54)
As [µg/L]	0.65 (0.48/0.81)	0.60 (0.48/0.75)	0.70 (0.55/0.95)	0.55 (0.45/0.85)
Mn [µg/L]	15.2 (8.93/22.1)	19.3 (12.5/27.7)	17.7 (11.6/24.2)	17.3 (10.8/28.3)
B [µg/L]	171 (139/213)	147 (105/194)	143 (116/187)	164 (128/197)
Li [µg/L]	1.64 (0.82/3.24)	1.48 (0.78/2.87)	1.33 (0.71/1.96)	1.35 (0.49/2.56)

**Table 5:** Concentrations of macro and trace elements in alpaca milk during four months postpartum, data are provided as median (1st quartile/3rd quartile), significant differences between the months are marked with different indices.

Milk	Day 6–30 (*n* = 17)	Day 31–60 (*n* = 16)	Day 61–90 (*n* = 16)	Day 91–120 (*n* = 12)
P [g/L]	1.21 (1.00/1.42)	1.15 (0.86/1.34)	1.15 (0.98/1.45)	1.11 (0.73/1.41)
Ca [g/L]	1.40 (1.23/1.55)^a^	1.33 (1.18/1.49)^a^	1.24 (1.02/1.55)^a,b^	1.11 (0.90/1.35)^b^
Mg [g/L]	0.16 (0.14/0.22)	0.15 (0.14/1.17)	0.15 (0.14/0.16)	0.13 (0.11/0.17)
Na [g/L]	0.50 (0.27/1.29)	0.42 (0.28/0.85)	0.46 (0.31/1.00)	0.52 (0.33/1.36)
K [g/L]	0.84 (0.78/1.11)^a^	0.96 (0.88/1.13)^a,b^	1.16 (1.13/1.28)^b^	0.98 (0.79/1.34)^a,b^
S [g/L]	0.60 (0.47/0.93)	0.50 (0.41/0.67)	0.49 (0.45/0.65)	0.54 (0.50/0.70)
Fe [mg/L]	1.16 (0.88/1.69)	1.59 (0.76/3.25)	1.33 (0.81/2.01)	1.08 (0.67/2.65)
Cu [mg/L]	0.25 (0.15/0.56)^a^	0.15 (0.13/0.17)^b^	0.13 (0.14/0.16)^b^	0.11 (0.08/0.14)^b^
Zn [mg/L]	3.16 (2.71/3.74)	3.07 (2.90/3.85)	2.94 (2.59/3.37)	3.19 (2.81/3.57)
Se [µg/L]	31.0 (19.9/43.6)^a^	20.2 (14.8/26.7)^b^	14.5 (8.68/26.3) ^b^	18.0 (11.9/33.4)^b^
Sr [mg/L]	0.77 (0.61/0.88)^a^	0.70 (0.66/0.84)^a^	0.61 (0.58/0.75)^a,b^	0.56 (0.41/0.63)^b^
Mo [µg/L]	6.50 (4.55/9.00)^a^	4.50 (3.50/7.65)^b^	2.80 (1.92/3.50)^c^	4.60 (2.80/5.98)^b^
Ba [µg/L]	514 (399/611)	753 (493/923)	641 (439/905)	449 (349/602)
Al [µg/L]	750 (204/950)	918 (698/1947)	1114 (560/1635)	607 (468/707)
Co [µg/L]	0.75 (0.40/1.15)	0.50 (0.30/1.10)	0.65 (0.40/1.10)	0.50 (0.30/0.70)
Ni [µg/L]	4.65 (3.58/5.92)	5.40 (4.50/8.70)	5.85 (4.65/8.00)	4.55 (3.65/6.05)
Tl [µg/L]	0.13 (0.11/0.17)	0.11 (0.08/0.17)	0.10 (0.08/0.16)	0.11 (0.08/0.14)
Pb [µg/L]	5.40 (2.02/7.85)^a^	9.80 (4.82/19.3)^b^	4.98 (3.24/10.1)^a^	3.00 (2.58/4.13)^c^
U [µg/L]	0.12 (0.09/0.14)	0.17 (0.14/0.19)	0.15 (0.13/0.19)	0.14 (0.12/0.20)
Cd [µg/L]	0.25 (0.08/0.40)	0.31 (0.12/0.44)	0.25 (0.13/0.39)	0.24 (0.18/0.32)
Sn [µg/L]	1.45 (0.78/3.45)^a^	3.10 (1.80/5.44)^b^	3.10 (1.88/3.70)^b^	3.40 (2.78/4.78)^b^
As [µg/L]	1.65 (0.98/1.90)^a^	1.80 (1.10/3.20)^a^	0.85 (0.70/2.05)^b^	0.60 (0.50/0.88)^b^
Mn [µg/L]	82.1 (46.2/118)^a^	131 (73.8/305)^b^	129 (77.6/257)^b^	76.5 (59.8/104)^a^
B [µg/L]	168 (147/200)	144 (129/180)	155 (122/185)	142 (135/167)
Li [µg/L]	12.8 (7.05/36.2)	15.6 (6.15/27.9)	11.9 (6.60/12.95)	12.60 (9.70/13.2)

**Table 6:** Comparison of concentrations of macro and trace elements in alpaca colostrum (samples from day 1 to day 4 postpartum) and milk (samples from day 11 to day 120 in lactation), data provided as median (1st quartile/3rd quartile), significant differences between the days are marked with different indices. For comparison upper concentration limits for drinking water (Austria) and concentration for llama and alpaca milk reported in the literature are provided.

Mineral	Colostrum (*n* = 77)	Milk (*n* = 61)	Water ^1^	Milk Llama (L) and Alpaca (A)
P [g/L]	1.85 (1.64/2.15)^a^	1.16 (0.92/1.38)^b^		A: 1.13 ± 0.16 ^2^, 0.98 ± 0.15 ^3^ L: 1.13 ± 0.16 ^4^
Ca [g/L]	1.71 (1.36/2.00)^a^	1.23 (1.07/1.35)^b^		A: 1.20 ± 0.16 ^2^, 1.38 ± 0.22 ^3^ L: 1.70 ± 0.18 ^4^
Mg [g/L]	0.29 (0.17/0.33)^a^	0.15 (0.14/0.17)^b^	0.05	A: 0.10 ± 0.02 ^2^, 0.13 ± 0.02 ^3^
Na [g/L]	0.37 (0.31/0.47)	0.42 (0.30/0.93)	0.20	A: 0.58 ± 0.28 ^2^, 0.20 ± 0.10 ^3^ L: 0.27 ± 0.18 ^4^
K [g/L]	1.26 (1.08/1452)^a^	0.98 (0.81/1.12)^b^		A: 1.61 ± 0.28 ^2^, 1.30 ± 0.19 ^3^ L: 1.20 ± 0.20 ^4^
S [g/L]	1.31 (0.92/1.83)^a^	5.40 (0.46/0.70)^b^	250^1^	A: 476 ± 112 ^3^, L: 425 ± 48.0 ^4^
Fe [mg/L]	0.79 (0,61/0.92)^a^	1.28 (0.77/2.01)^b^	0.20	L: 0.65 ^4^
Cu [mg/L]	0.48 (0.43/0.62)^a^	0.14 (0.11/0.19)^b^	2.00	L: 0.11 ^4^
Zn [mg/L]	5.12 (3.44/6.37)^a^	3.07 (2.80/3.57)^b^		L: 4.19 ± 0.95 ^4^
Se [µg/L]	117 (81.1/173)^a^	21.4 (13,3/33.5)^b^	10.0	
Sr [mg/L]	1.15 (0.94/1.62)^a^	0.66 (0.58/0.80)^b^		
Mo [µg/L]	8.60 (5.30/14.8)^a^	4.50 (2.90/6.86)^b^		
Ba [µg/L]	656 (410/969)	582 (418/783)		L: 278 ^4^
Al [µg/L]	42.0 (26.1/62.8)^a^	762 (598/1362)^b^	200	L: 416 ^4^
Co [µg/L]	2.59 (0.99/4.34)^a^	0.50 (0.33/1.10)^b^		
Ni [µg/L]	7.30 (5.94/8.47)^a^	5.25 (4.35/6.84)	20.0	
Tl [µg/L]	0.11 (0.07/0.16)	0.12 (0.10/0.19)		
Pb [µg/L]	1.00 (0.69/1.62)^a^	4.81 (3.00/9.77)^b^	40.0	
U [µg/L]	0.04 (0.01/ 0.10)^a^	0.17 (0.10/0.50)^b^	15.0 ^1^	
Cd [µg/L]	0.06 (0.00/0.16)^a^	0.30 (0.10/0.35)^b^	5.00 ^1^	
Sn [µg/L]	0.80 (0.40/1.39)^a^	2.93 (1.49/4.33)^b^		
As [µg/L]	0.60 (0.49/0.82)^a^	1.11 (0.75/1.88)^b^		
Mn [µg/L]	17.2 (11.6/24.4)^a^	93.7 (69.2/209)^b^	50.0 ^1^	L: 71.0 ^4^
B [µg/L]	159 (126/186)	150 (132/180)	1000 ^1^	
Li [µg/L]	1.31 (0.78/1.86)^a^	12.5 (6.85/20.0)^b^		

^1^ Ordinance of the Austrian Federal Minister for Social Security and Generations on the Quality of Water for Human Consumption (Drinking Water Ordinance-TWV) StF: BGBl. II Nr. 304/2001 [CELEX-Nr.: 398L0083] of 10.01.2018. ^2^ Martini [17]. ^3^ Chad [18]. ^4^ Morin [7].

## Data Availability

Data available on request. The data presented in this study are available on request from the corresponding author.
